# Elongation of the inferior rectus tendon with fascia lata graft for large vertical squint angles in patients with Graves’ orbitopathy

**DOI:** 10.1007/s00417-022-05696-5

**Published:** 2022-05-19

**Authors:** Julia Prinz, Kathi Hartmann, Filippo Migliorini, Karim Hamesch, Peter Walter, Matthias Fuest, David Kuerten

**Affiliations:** 1 Department of Ophthalmology, RWTH Aachen University, 52074 Aachen, Germany; 2 Department of Orthopedics, RWTH Aachen University, 52074 Aachen, Germany; 3 Department of Gastroenterology and Hepatology, RWTH Aachen University, 52074 Aachen, Germany

**Keywords:** Tendon elongation, Fascia lata, Graves’ disease, Strabismus

## Abstract

**Purpose:**

To investigate the use of fascia lata (FL) grafts for inferior rectus muscle (IRM) tendon elongation in patients with large vertical squint angles with Graves’ orbitopathy (GO).

**Methods:**

In this retrospective study, we included a consecutive series of 20 eyes of 13 patients with GO who underwent IRM tendon elongation with FL. Orthoptic and ophthalmologic examinations including measurement of the head posture, the extent of deviation in primary position (PP), elevation, motility, and binocular diplopia at the tangent of Harms were conducted preoperatively and after a mean postoperative time of 10.8 (5.0–35.0) months in all patients.

**Results:**

The mean total repositioning distance was 9.3 ± 3.6 (3.5–16.0) mm. Postoperatively, we found a significant increase in elevation (5.4 ± 2.4 vs. 2.7 ± 2.4 mm preoperatively, *p* = 0.011). A significant reduction in vertical squint angle (2.8 ± 3.7 vs. 20.2 ± 18.8 Δ preoperatively, *p* = 0.004), chin elevation (2.3 ± 3.7 vs. 12.9 ± 6.3° preoperatively, *p* < 0.001), extorsion in PP (0.1 ± 3.8 vs. 8.4 ± 7.8° preoperatively, *p* = 0.002), and in elevation (1.8 ± 4.8 vs. 11.1 ± 10.9° preoperatively, *p* = 0.004) occurred postoperatively. A mean dose–effect relation of 2.6 ± 2.9 Δ/mm was calculated. Postoperatively, the lower eyelid retraction was significantly increased (1.5 ± 1.4 vs. 0.4 ± 0.5 mm preoperatively, *p* = 0.005).

**Conclusion:**

IRM tendon elongation with FL is a feasible and effective procedure without relevant risk for surgery-related complications.


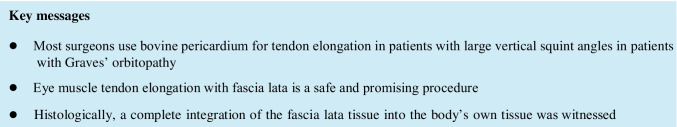


## Introduction

Graves’ orbitopathy (GO) is the most common extrathyroidal manifestation of Graves’ disease with an incidence of 16/100,000 in females and 2.9/100,000 in males [1]. Ocular motility restrictions in patients with GO are mainly attributed to the fibrosis of eye muscles [2]. The affected muscles lose extensibility, while their contractility is slightly restricted [3]. The inferior rectus muscle (IRM) is most commonly affected, resulting in limited elevation of the eye and hypotropia with vertical diplopia and extorsion on upgaze [4]. Treatment by the simple recession of the IRM is limited to a maximum distance of about 8 mm due to the necessity to maintain a sufficient arc of contact [5], whereas treatment via muscle surgery with tendon elongation allows a correction of larger squint angles [6]. In previous studies, different materials have been used for tendon elongation, such as homologous scleral grafts, polytetrafluoroethylene (Goretex), silicone, or bovine pericardium [7–9]. In 2011, Esser and Eckstein reported good long-term results of large squint angle correction by tendon elongation with bovine pericardium in patients with GO [9]. However, other authors described severe complications after using bovine pericardium in different indications [10].

Fascia lata (FL) is the fibrous tissue sheath enveloping the muscle groups of the thigh [11]. Histologically, FL consists of a collagen matrix with fibroblasts [11]. Its suitability for grafting is attributed to the relative acellularity and low nutritional requirements [11]. FL is characterized by its tensile strength and pliability [12]. Its fibrous, sheet-like appearance allows it to be cut and shaped for different surgical and reconstructive demands [11]. In addition, FL is homologous tissue which shows no risk of foreign body reaction and only a low risk of infection [12]. Due to these advantages, FL grafts have been used in different surgical specialties, including urologic [13], orthopedic [14], cardiac [15], and general surgery [16]. In ophthalmic surgery, Payr pioneered the use of FL in the correction of congenital ptosis in 1909 [17]. Since then, FL has been used for the treatment of different ophthalmic conditions, such as scleromalacia perforans [18], cicatricial entropium, thyroid lid retraction, and orbital implant extrusion [11].

In this study, we evaluated the use of FL for IRM tendon elongation in patients with large vertical squint angles associated with GO.

## Material and methods

**Fig. 1:**
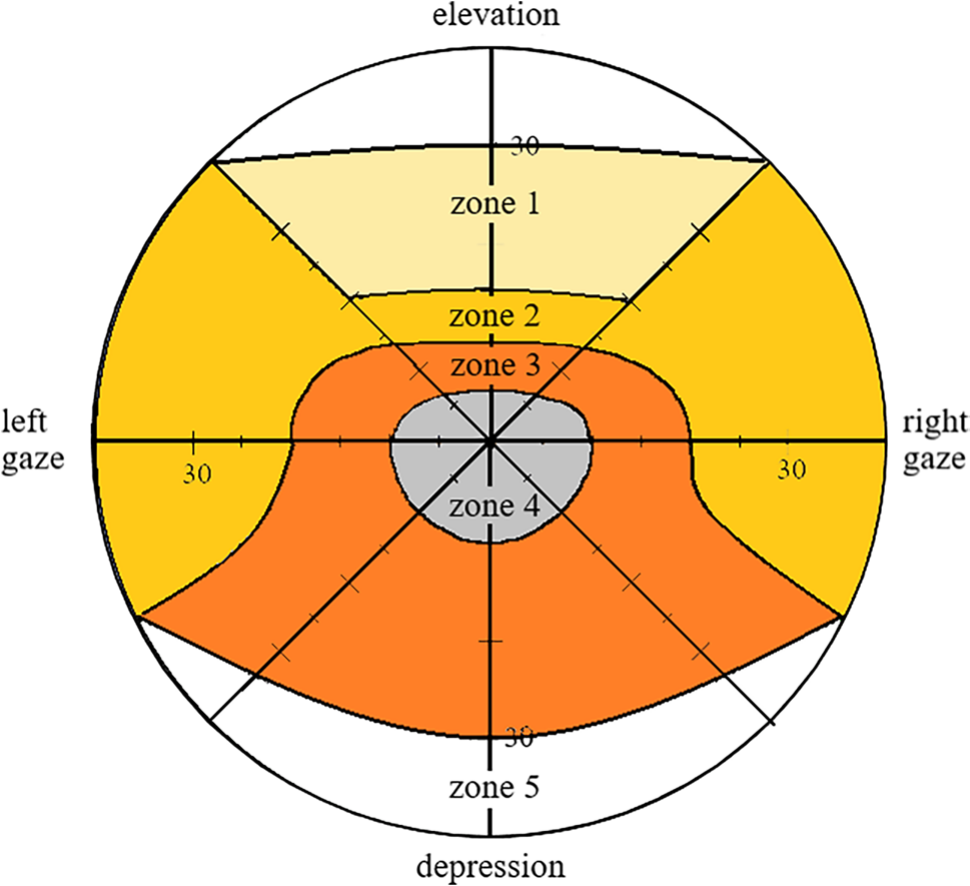
Schema of Haase and Steinhorst to evaluate binocular diplopia according to the recommendation of the German Ophthalmological Society (modified after Gramberg-Danielsen et al. [23])

In this retrospective study, we included a consecutive series of 20 eyes of 13 patients who underwent IRM tendon elongation with FL between October 2011 and June 2015. Only patients with GO and large vertical squint angles potentially requiring a recession of the IRM of more than 4 mm and a follow-up period of at least 5 months were included. All patients were treated at the Department of Ophthalmology, RWTH Aachen University. Orthoptic and ophthalmologic examinations were conducted preoperatively and after a mean postoperative time of 10.8 (5.0–35.0) months in all patients. The examinations included measurement of the extent of deviation in primary position (PP) by alternate prism cover test (APCT) at far fixation with best-corrected visual acuity (BCVA). The prism was placed in front of the eye with worse motility to ensure the measurement of the primary angle. In cases of poor vision in one eye, we applied the Krimsky test [19]. In addition, each examination comprised measurement of a head posture by a hand-held goniometer at far fixation with BCVA and a test for binocular function (Bagolini striated glasses test [20] or Lang I/II stereo test [21]). Measurement at the tangent of Harms [22] was performed in the six fields of action of the eye. The evaluation of binocular diplopia was performed according to the schema of Haase and Steinhorst (Fig. 1) [23]. The motility of each eye was evaluated regarding over- or underactions in the main fields of action of all six eye muscles. The elevation was measured as the monocular excursion at the inferior limbus from PP to maximum elevation in mm. The distance between the inferior limbus and the lower eye lid margin was considered as lower eye lid retraction, measured with head straight. Additionally, we collected data on age, sex, and ocular history.

The retrospective case series was approved by the medical ethics committee of the RWTH Aachen (EK 411/21).

### Surgical technique

**Fig. 2:**
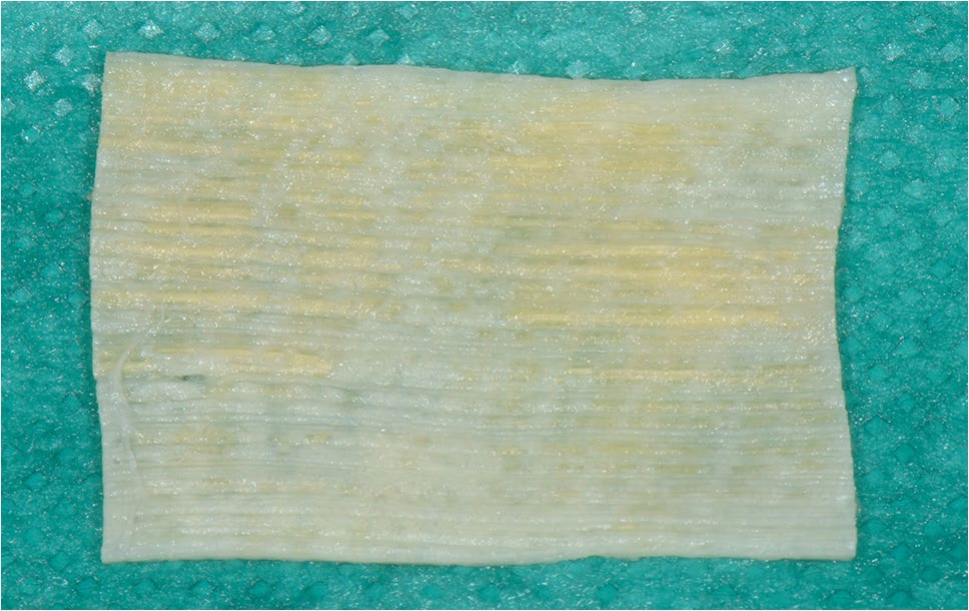
Non-vital fascia lata (FL) Tutograft made from allogenic FL at size 20 × 30 mm. First, the graft is rehydrated in a balanced salt solution according to the manufacturer’s recommendation. Then, the graft is cut to fit the surgeon’s demands

**Fig. 3:**
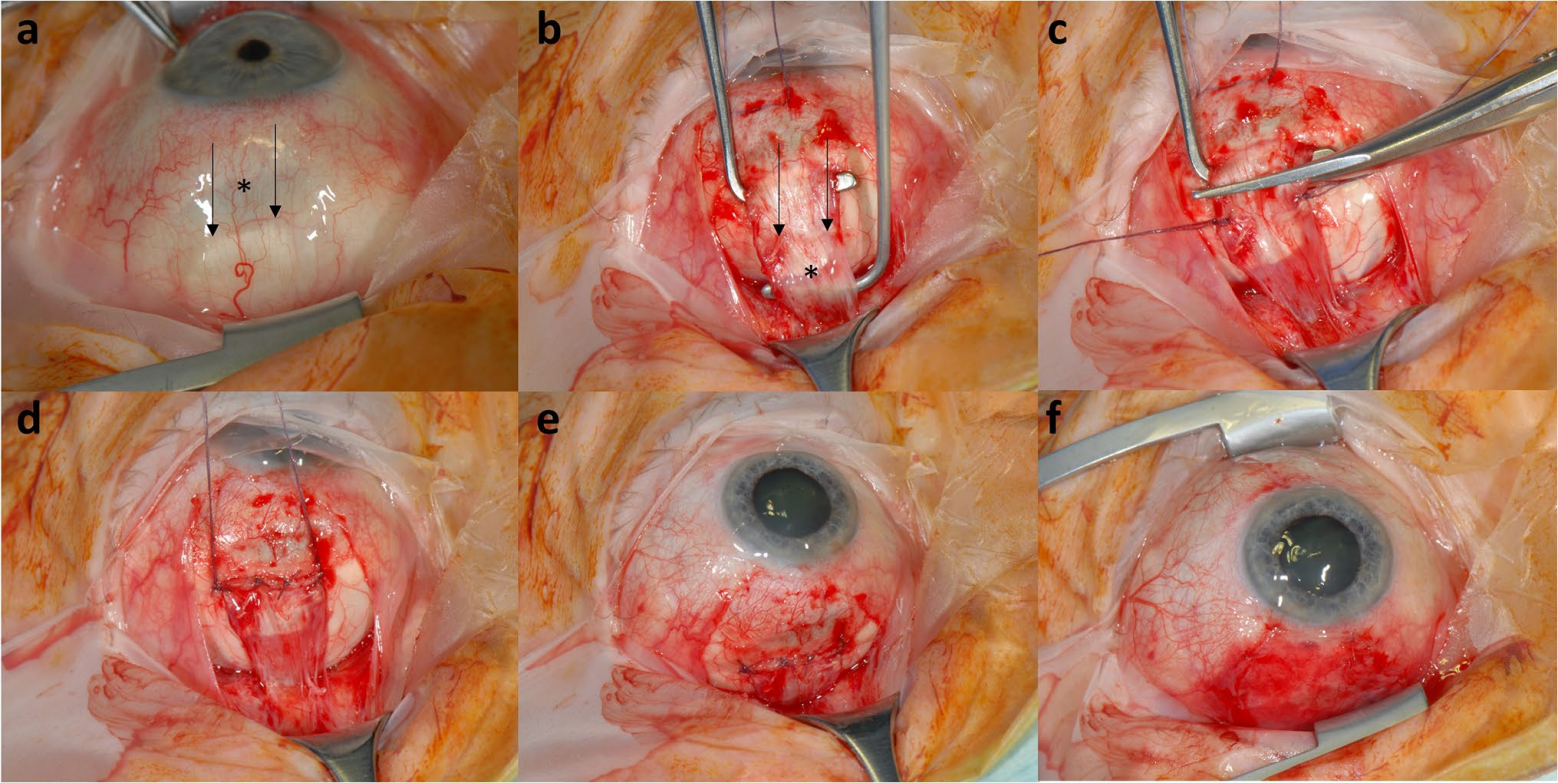
(**a**) Postoperative appearance in a patient 11 months after inferior rectus muscle (IRM) tendon elongation with fascia lata (FL). The FL was visible through the conjunctiva about 3 mm behind the physiological insertion (arrows). The sclera behind the original IRM insertion (*) appeared thin with the underlying uvea visible. (**b**) In this patient receiving revision surgery due to a postoperative vertical squint angle, the conjunctiva was incised and the Tenon’s capsule around the IRM was dissected. The transition between the IRM and FL graft was hardly visible (arrows). The area between the arrows and the star (*) represents the overlapping FL graft. Then, the muscle was disinserted (**c**). Due to the over-effect in this patient, the IRM tendon and FL were placed behind the previous insertion and sutured to the sclera (**d**). Finally, the sutures were shortened (**e**), and the conjunctiva was sutured (**f**). When implanted, the FL tendon is significantly thicker than the muscle’s tendon. Over time, the FL graft becomes thinner, vascularized, and macroscopically can hardly be distinguished from the endogenous tendon without losing its stability

The exact surgical procedure depended on the patients’ vertical deviation, head posture, and motility. As the affected muscles show both reduced extensibility and slightly restricted contractility, an exact preoperative surgical dose calculation for the elongation of the IRM was often not possible. Intraoperatively, the passive ocular motility after disinsertion of the IRM was usually still restricted due to connective tissue contracture in patients with GO. While fixating the FL graft, the motility was tested again and must not be worse; otherwise, the FL graft length was adjusted. The aim of each surgery was the elimination of diplopia in PP and a reduction of head posture. The full extent of pre- and postoperative squint angle in PP was considered to calculate the dose effect relation. All procedures in this study were performed by the same experienced surgeon (K.H.). Surgery was performed under general anesthesia. First, an intraoperative forced duction test was conducted. A perilimbal conjunctival incision with radial relaxing incisions was performed at the inferior conjunctiva. A muscle hook secured the IRM at its insertion. Then, the Tenon’s capsule around the IRM was dissected. The IRM was held by a muscle clamp and then disinserted. As a graft for elongation, we used allogenic Tutoplast FL (Bess Medizintechnik GmbH, Berlin, Germany, Fig. 2). The FL graft was first rehydrated in a balanced salt solution (Alcon BSS, Alcon Pharma GmbH, Freiburg, Germany), then cut to an estimated length. The posterior end of the graft was sutured to the detached end of the IRM using absorbable Vicryl 5–0 (Ethicon, Johnson & Johnson Medical GmbH, Norderstedt, Germany) suture with an overlap of a few mm (Fig. 3b, between the arrows and the star). A corner suture was placed at both borders perpendicular to the direction of the fibers of the FL to avoid a rupture of the graft.

When the IRM was very fibrotic and the muscle clamp could not be used, the posterior end of the FL graft was sutured to the IRM directly behind the insertion without previous disinsertion of the IRM. Then, the IRM was disinserted afterward. The graft length was then measured from the sutures. As the sutures should not be visible through the conjunctiva postoperatively, the anterior end of the graft was sutured to the sclera about 3 mm behind the physiological insertion with 2 corner sutures perpendicular to the direction of the fibers of the FL. The surplus FL was then cut off with an excess of 2 mm FL left so that the graft cannot tear out. In cases of previous recession surgery of the IRM, the insertion of the elongated tendon was advanced and the original tendon elongated. Thus, we aimed at enhancing the arc of contact while weakening the muscle. Finally, the conjunctiva was sutured with Vicryl 9–0 (Ethicon, Johnson & Johnson Medical GmbH, Norderstedt, Germany).

**Fig. 4:**
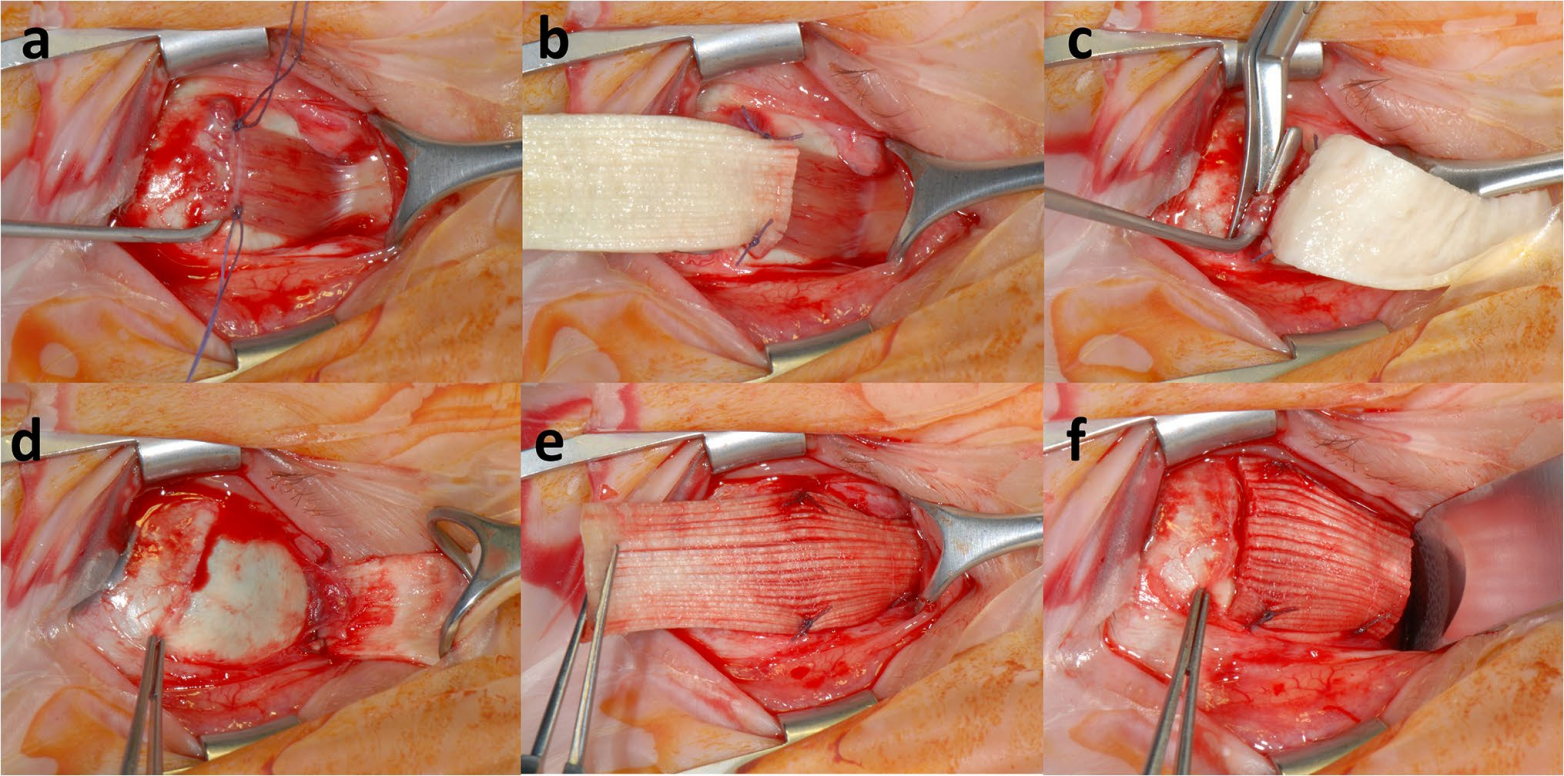
Exemplary surgical procedure of a medial rectus tendon recession and elongation with fascia lata (FL) graft in a patient with abducens nerve palsy. Following a perilimbal incision of the conjunctiva with two relaxing incisions, the muscle can be held with a muscle clamp. In this case, the muscle was very fibrotic and the muscle clamp could not be used. Therefore, a corner suture (Vicryl 5–0) was placed at both borders of the muscle close to its insertion (**a**) and at the FL graft perpendicular to the direction of the fibers of the FL to avoid a rupture of the graft (**b**). Thus, the posterior end of the FL graft was sutured to the muscle directly behind the insertion without previous disinsertion of the muscle (**b**). The muscle was disinserted afterward (**c**). Then, we tested for ocular motility with the muscle disinserted and decided on the final FL graft length (**d**). Two Vicryl 5–0 sutures were placed in the sclera 3 mm behind the physiological insertion, avoiding the sutures to be visible through the conjunctiva postoperatively. The graft was then sutured to the sclera with the sutures perpendicular to the direction of the fibers of the FL (**e**). Finally, the surplus FL was cut off with an excess of 2 mm FL to avoid that the graft might tear out (**f**)

As no further intraoperative photographs during IRM elongation with FL were available, an exemplary surgical procedure of a medial (not IRM) rectus tendon recession and elongation with FL in a patient with abducens nerve palsy is displayed in Fig. 4 for better visualization.

### Statistical analysis

Statistical analysis was performed using the Statistical Package for Social Sciences (IBM SPSS Statistics for Windows, Version 25, Armonk, NY: IBM Corp.). All values are displayed as mean ± standard deviations (SD). Means for continuous variables were compared using independent-group T-tests when the data were normally distributed; otherwise, the Mann–Whitney *U* test was used. The Kolmogorov–Smirnov test was used to verify that data was normally distributed. Chi-square test and Fisher’s exact test were used for categorical variables. For multiple testing, Bonferroni adjustment was calculated. A *p*-value of < 0.05 was considered statistically significant.

## Results

### Patients’ characteristics

Our study included 10 right (50.0%) and 10 left (50.0%) eyes of 13 patients (7 bilateral surgeries). The mean age of all patients was 57.7 ± 12.4 (38.0–82.0) years. Two patients (15.4%) were male. In all patients, GO was confirmed by clinical symptoms, blood tests, orthoptic status, and by computed tomography (mean duration of GO to surgery was 24.7 (6.0–64.0) months). Preoperatively, thyroid and orthoptic status were stable in all patients for at least 6 months (mean preoperative TSH-value 2.2 (0.5–4.0) mlU/L). Eleven patients reported previous steroid therapy, 3 patients had a previous radioiodine therapy, and 1 patient previously underwent orbital decompression surgery. Previous IRM tendon recession of 7 mm was performed in a patient (both eyes) with no sufficient effect. A bilateral FL elongation was performed on bilateral asymmetrically contracted IRM if a head posture (chin lift) had to be assumed after prism adjustment on the more affected side.

**Fig. 5:**
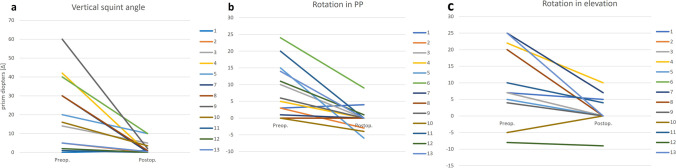
(**a**) Pre- and postoperative full extent of vertical squint angle in prism diopters (Δ) for all patients (1–13) in primary position (PP). To better visualize the extent of vertical squint angle, all angles are illustrated as positive values. (**b**) Pre- and postoperative full extent of rotation in prism diopters for all patients (1–13) in PP and in the elevation (**c**). Extorsion is given in positive, intorsion in negative values

Neither intra- nor postoperative surgery-related complications occurred. The IRM tendon was elongated with a mean FL graft length of 6.6 ± 3.0 (2.0–13.0) mm. The mean recession distance was 2.7 ± 1.3 (0.0–4.0) mm. In the patient with previous IRM tendon recession, no additional recession was performed. The mean total repositioning distance was 9.3 ± 3.6 (3.5–16.0) mm. A significant increase in elevation occurred postoperatively (5.4 ± 2.4 mm) compared to preoperatively (2.7 ± 2.4 mm, *p* = 0.011, Table 1). We found a significant reduction in vertical squint angle (preoperatively 20.2 ± 18.8 Δ, postoperatively 2.8 ± 3.7 Δ, *p* = 0.004, Fig. 5) and head posture (chin elevation preoperatively 12.9 ± 6.3°, postoperatively 2.3 ± 3.7°, *p* < 0.001) at the postoperative visit compared to preoperatively (Table 1). A mean dose effect relation of 2.6 ± 2.9 Δ/mm was calculated. Both in PP and in elevation, a significant reduction of extorsion occurred postoperatively (PP: preoperatively mean extorsion 8.4 ± 7.8°, postoperatively 0.1 ± 3.8°, *p* = 0.002, elevation: preoperatively mean extorsion 11.1 ± 10.9°, postoperatively 1.8 ± 4.8°, *p* = 0.004, Fig. 5). Postoperatively, the lower eyelid retraction was significantly increased (preoperatively 0.4 ± 0.5 mm, postoperatively 1.5 ± 1.4 mm, *p* = 0.005). The exact pre- and postoperative mean, minimum, maximum, and SD values are displayed in Table 1.

**Table 1:** Pre- and postoperative mean, minimum, maximum values, and standard deviations of the elevation distance in mm (measured as monocular excursion at the inferior limbus from primary position to maximum elevation), the vertical squint angle in prism diopters (Δ), the chin elevation in degrees (°) in primary position as a head posture, the rotation in ° in primary position and in elevation, and the retraction of the lower lid as measured as the distance between the inferior limbus and the lower eyelid margin

	Preoperative				Postoperative				*p*-value
	Mean	Min	Max	SD	Mean	Min	Max	SD	
Elevation [mm]	2.7	− 1.0	6.5	2.4	5.4	2.0	8.8	2.4	**0.011**
Vertical squint angle [Δ]	20.2	0.0	60.0	18.8	2.8	0.0	10.0	3.7	**0.004**
Chin elevation [°]	12.9	7.0	30.0	6.3	2.3	0.0	10.0	3.7	** < 0.001**
Rotation in PP [°]	Ex 8.4	In 2.0	Ex 24.0	7.8	Ex 0.1	In 6.0	Ex 9.0	3.8	**0.002**
Rotation in elevation [°]	Ex 11.1	In 8.0	Ex 25.0	10.9	Ex 1.8	In 9.0	Ex 10.0	4.8	**0.004**
Lower lid retraction [mm]	0.4	0.0	1.8	0.5	1.5	0.0	4.0	1.4	**0.005**

Postoperatively, we found a significant increase in the elevation distance (*p* = 0.011), a significant reduction of the vertical squint angle (*p* = 0.004), and a significant reduction in the angle of chin elevation (*p* < 0.001) compared to preoperative values. Postoperatively, the rotation showed a significant tendency toward intorsion compared to preoperatively both in primary position (*p* = 0.002) and in elevation (*p* = 0.004). The retraction of the lower lid increased significantly from pre- to postoperatively (*p* = 0.005)

*Ex* extorsion, *In* intorsion, *Min* minimum, *Max* maximum, *SD* standard deviation, *PP* primary position

According to the schema of Haase and Steinhorst [23], binocular diplopia was evaluated in 5 zones (Fig. 1). Zones 3 and 4 represent the main field of vision. The data from 1 patient was missing as the patient was lost to follow-up. Preoperatively, binocular diplopia was reported by 11 patients in zones 1 to 3 and by 10 patients in zones 4 to 5 (Table 2). A patient showed suppression of one eye in all 5 zones. Postoperatively, binocular diplopia was diagnosed in 10 patients in zones 1, 9 patients in zone 2, 6 patients in zone 3, 3 patients in zone 4, and 5 patients in zone 5. No patient showed suppression of either eye postoperatively. In the main field of vision (zones 3 and 4), binocular diplopia was reported by 91.7% of all tested patients preoperatively and by 41.7% postoperatively (*p* = 0.053). Table 2 shows the numbers of patients reporting single binocular vision, diplopia, and suppression pre- and postoperatively.

**Table 2:** Pre- and postoperative results of single binocular vision (SBV) in zones 1 to 5 according to the schema of Haase and Steinhorst [23] in 12 patients

Zone	Preoperative				Postoperative			
	SBV	Dp	Sup	Missing	SBV	Dp	Sup	Missing
1	0	11	1	1	2	10	0	1
2	0	11	1	1	3	9	0	1
3	0	11	1	1	6	6	0	1
4	1	10	1	1	9	3	0	1
5	1	10	1	1	7	5	0	1

The data from 1 patient was missing. In zone 4 (primary position), the number of patients with SBV changed from 1 to 9 patients, while binocular diplopia (Dp) was reported by 10 patients preoperatively and only by 3 patients postoperatively. A patient showed a suppression (Sup) of one eye preoperatively. In zone 5 (depression), the number of patients reporting SBV or Dp changed from 1 patient preoperatively to 7 patients postoperatively and from 10 to 5 patients, respectively

### Histologic results

**Fig. 6:**
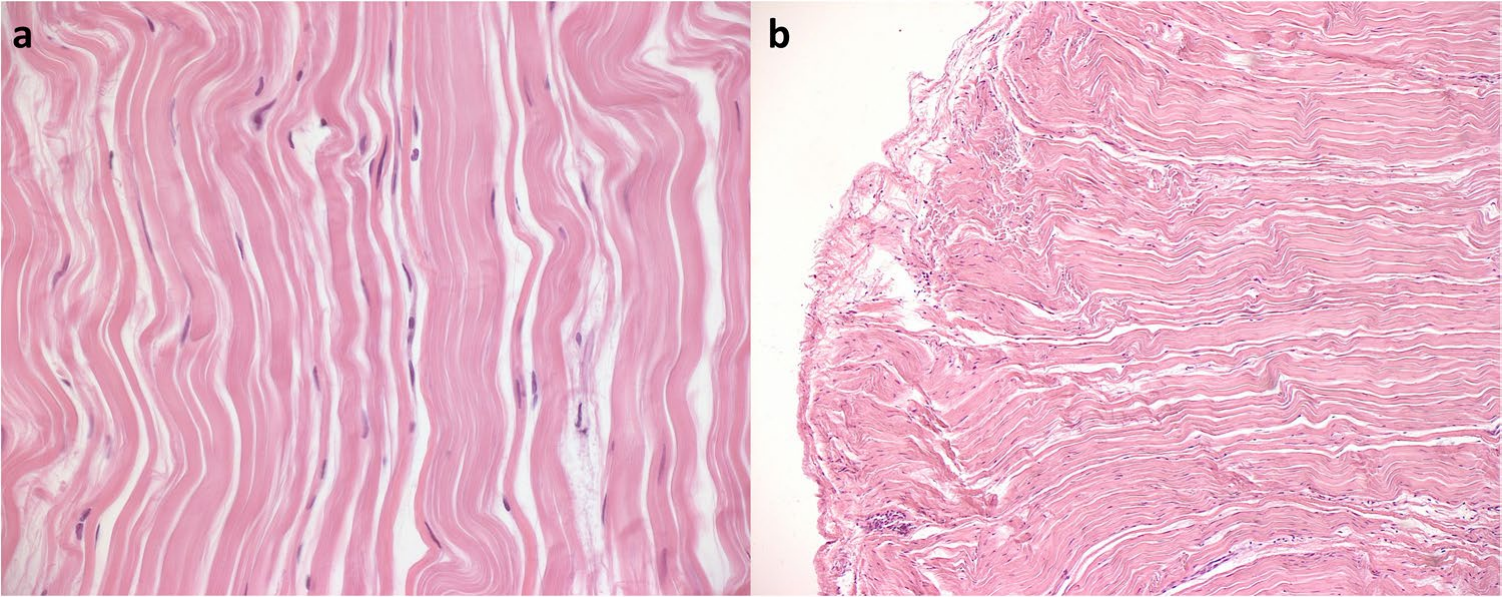
The histological findings of the fascia lata (FL) graft 11 months postoperatively. At high-magnification (**a**), the preparation showed parallel collagen fibers aligned like in tendon tissue. At the outer edge of the preparation in lower magnification (**b**), a suture granuloma was found

Eleven months postoperatively, revision surgery with further IRM tendon and lower eyelid elongation was necessary for a patient with a large postoperative vertical squint angle and prominent lower eyelid retraction. Intraoperatively, we gained a piece of the earlier implanted FL graft for histological preparation (Fig. 6). The histopathological result confirmed no remnants of the FL graft. Microscopically, the hematoxylin and eosin-stained preparation showed parallel collagen fibers aligned like in tendon tissue. At the outer edge of the preparation, a suture granuloma with giant cells was diagnosed.

## Discussion

In this study, IRM tendon elongation with FL was evaluated in patients with GO. In all patients, a simple surgical recession of the IRM was deemed unlikely to achieve satisfying results. After surgery (tendon elongation via FL), we found a significant reduction in the vertical squint angle and head posture. In addition, the monocular elevation excursion increased significantly. A significant tendency toward intorsion occurred after surgery. To our knowledge, this is the first study addressing the efficacy of eye muscle tendon elongation with FL.

### Surgical effect

Our results showed a postoperative squint angle of 10 Δ or less in all patients. A mean dose effect relation of 2.6 ± 2.9 Δ/mm was calculated in our study. In 2011, Esser et al. published their results on IRM tendon elongation with bovine pericardium [9]. They included 10 patients with GO and large vertical squint angles. A dose effect relation of 2.0°/mm, which is equivalent to 3.5 Δ/mm, has been reported [9]. The same authors investigated medial rectus elongation with bovine pericardium in GO patients. A dose effect relation of 1.8 ± 0.6 Δ/mm was achieved [3]. Wipf et al. performed medial rectus tendon elongation with bovine pericardium in 5 patients with GO and reported a dose–effect relation of 3.6 Δ/mm [24]. Generally, dose–effect relations for the recession of the medial rectus muscle are lower than for the IRM [24]. Overall, the dose–effect relation of tendon elongation with bovine pericardium showed high variability, which might be attributable to the different muscles and pathologies reported in previous studies [6]. Furthermore, the bovine pericardium was described to be slightly elastic [25]. In comparison, FL is a very fibrous tissue [11]. When directly comparing the macroscopic appearance, FL is much thicker and seems to be less flexible and elastic than bovine pericardium. Thus, we hypothesize that the relative elasticity of bovine pericardium might explain the high variability and higher dose effect relations of bovine pericardium compared to our results with FL.

The primary action of the IRM is depressing the eye, while secondary and tertiary actions are the adduction and extorsion of the eye [26]. Thus, the tendency toward intorsion after surgery is caused by the fact that tendon elongation counteracts the preoperative excessive extorsion by the fibrotic muscle.

### Side effects

No surgery-related intra- or postoperative complications, especially no allergic, infectious or foreign body reactions requiring treatment, were reported.

In a previous study, conjunctival penetration of non-absorbable sutures of the implant has been reported after medial rectus tendon elongation in patients with GO [3]. Therefore, Oeverhaus et al. previously recommended absorbable sutures for eye muscle elongations [3]. Using absorbable sutures, no conjunctival penetration was recorded in our patients. The absorbable sutures are degraded by a slight inflammatory reaction [27], which can be seen in the histological images in Fig. 6. Non-absorbable sutures are not required because the FL, fixed with two absorbable corner sutures, behaves like the endogenous tendon and attaches completely to the sclera and the IRM.

We found a significant increase in lower lid retraction postoperatively. Similarly, Pacheco et al. reported the development of lower lid retraction in 94% of patients after the IRM recession [28]. The authors attributed this undesired effect to the close anatomical position of the IRM to the lower lid retractors and the intermuscular membranes.

### Biocompatibility

Previously, non-ophthalmic studies reported multiple benefits of FL grafts, such as a very low risk of infection, displacement, or extrusion [29]. To date, no reports of transmission of viral or prion diseases by FL have been published [29]. Generally, the allogenic FL Tutoplast graft is of human origin and therefore is not a xenogeneic graft.

Previous reports highlight the possibility of complete integration of FL tissue transplants into the body’s own tissue [29]. The same observation was confirmed by our histological examination, which showed tendon-like tissue instead of FL graft 11 months after implantation of the graft (Fig. 6). Due to our and previously published findings, FL seems to be particularly well suited for tendon elongation, especially as the fibers are longitudinally orientated and can thereby easily be sutured to the preexisting tendon/muscle.

The lack of a control group and the retrospective design of our study pose limitations for the analysis of IRM tendon elongation with FL. A mean dose effect relation of 2.6 ± 2.9 Δ/mm was calculated in our study. However, given the limited sample size, a precise recommendation on the exact surgical dose is not possible yet. In cases of a large preoperative elevation deficiency, chin elevation, or pronounced pseudo-Dalrymple’s sign, we recommend an FL graft length of at least 8 mm. Moreover, we recommend an intraoperative ocular motility test after disinsertion of the IRM. When the FL graft is fixated, the motility should not become worse. As previous studies revealed a postoperative drift toward overcorrections in patients with GO [30], we excluded patients with less than 5 months of follow-up to correct for postoperative drift. However, in a separate analysis, no significant differences in the 2 excluded patients with shorter follow-up were recorded. We are currently working on a larger-cohort analysis with an extended follow-up to hopefully provide dose–effect relations and to investigate whether a postoperative drift might occur in the long term.

Altogether, according to the main findings of the present study, eye muscle tendon elongation with FL is a safe and promising procedure without relevant risk for surgery-related severe complications. As GO is characterized by contracture and inflammatory paresis of the IRM with the contracture predominating, recessions of more than 4 mm can already lead to a limited depression of the eye. We therefore recommend the use of FL grafts in cases which potentially require a recession of the IRM of more than 4 mm. Using FL for tendon elongation seems to be reasonable, especially when the surgery is combined with lid surgery requiring FL. In those cases, only one graft is needed, which reduces surgery costs. However, future studies are warranted to directly compare the outcomes of tendon elongation with FL to those with bovine pericardium or other materials.
